# A Novel Adaptive H∞ Filtering Method with Delay Compensation for the Transfer Alignment of Strapdown Inertial Navigation Systems

**DOI:** 10.3390/s17122753

**Published:** 2017-11-28

**Authors:** Weiwei Lyu, Xianghong Cheng

**Affiliations:** 1 School of Instrument Science & Engineering, Southeast University, Nanjing 210096, China; lvww0220@seu.edu.cn; 2 Key Laboratory of Micro-Inertial Instrument and Advanced Navigation Technology, Ministry of Education, Southeast University, Nanjing 210096, China

**Keywords:** strapdown inertial navigation system (SINS), transfer alignment, time delay, robustness factor, adaptive H∞ filter

## Abstract

Transfer alignment is always a key technology in a strapdown inertial navigation system (SINS) because of its rapidity and accuracy. In this paper a transfer alignment model is established, which contains the SINS error model and the measurement model. The time delay in the process of transfer alignment is analyzed, and an H∞ filtering method with delay compensation is presented. Then the H∞ filtering theory and the robust mechanism of H∞ filter are deduced and analyzed in detail. In order to improve the transfer alignment accuracy in SINS with time delay, an adaptive H∞ filtering method with delay compensation is proposed. Since the robustness factor plays an important role in the filtering process and has effect on the filtering accuracy, the adaptive H∞ filter with delay compensation can adjust the value of robustness factor adaptively according to the dynamic external environment. The vehicle transfer alignment experiment indicates that by using the adaptive H∞ filtering method with delay compensation, the transfer alignment accuracy and the pure inertial navigation accuracy can be dramatically improved, which demonstrates the superiority of the proposed filtering method.

## 1. Introduction

A strapdown inertial navigation system (SINS) needs to complete initial alignment before it begins to work [1]. The purpose of the initial alignment is to make the SINS’s mathematical platform be consistent with the selected navigation coordinate system [2], and consequently a suitable initial navigation coordinate system can be established. In the process of initial alignment, high alignment accuracy and fast alignment speed are two important factors that directly affect the practical performance of the SINS [3].

As an important method of initial alignment, transfer alignment has been widely used in various applications such as aircraft, ships and vehicles, because of its speed and high accuracy [4]. In [5,6] an innovative transfer alignment method based on parameter identification UKF and an innovative transfer alignment method based on federated filter were respectively designed for an airborne distributed Position and Orientation System (POS). Shortelle and Graham [7] conducted a series of rapid transfer alignment experiments on F-16 fighters. In [4] a method based on iterative calculation for fast and high-accuracy transfer alignment between master SINS (M-SINS) and slave SINS (S-SINS) for ships was proposed. Lim and Lyou [8] designed an error compensation method based on state augmentation and robust state estimation to reduce the transfer alignment errors on ships. The S-SINS makes full use of accurate attitude, velocity and position information of M-SINS to set its initial parameters, and it can complete the alignment in a short period of time. In the whole navigation system, M-SINS and S-SINS are in different spatial positions. When M-SINS transmits the baseline information to S-SINS, there inevitably exists a time delay. The time delay between M-SINS and S-SINS can lead to large alignment errors during the period of transfer alignment [9]. Liu et al. [10] analyzed the effect of time delay on velocity matching transfer alignment under different maneuvering conditions. In [11] the delay time was extended to the system state model and it could be estimated through the Kalman filter, but this method increases the filtering computation and the estimation accuracy is limited, so it is necessary to develop a new method to accurately estimate and compensate the time delay.

Transfer alignment needs to use filtering technology to estimate online the S-SINS’s misalignment angles, and the attitude matrix can be effectively corrected at the end of alignment [12,13,14,15,16,17,18]. When the base is moving, there exist interference factors such as external noises or motion disturbances, which can decrease the accuracy of a S-SINS’s transfer alignment [19,20].

The traditional Kalman filter requires an accurate system mathematical model and detailed information on the statistical properties of the signal [19,20,21,22,23], but in practical applications, the statistical characteristics of signals are not accurate, and the system model itself also has some indetermination, so there is uncertainty in the system [20,21,22,24,25,26,27]. When the uncertainty reaches a certain extent, the estimation accuracy of the Kalman filter will be reduced, and Kalman filter may even have divergence problems in serious cases [20]. Comparatively, the H∞ filter is not based on any assumption about the frequency spectrum characteristics of the signal, and it has better robustness than the Kalman filter [20,22,23,24,25,26,27,28,29,30,31,32]. In the H∞ filter, the H∞ norm is introduced to the filtering problem, and a filter is designed to ensure the minimum norm from disturbance input to filter error output [20,23]. The estimation errors of states can be minimized in the worst cases of disturbance. The H∞ filter can be considered as the optimal filter when the system sustains serious disturbances, and filtering robustness is its most prominent characteristic [30,31,32]. Due to the above advantages, the H∞ filter is more suitable than the Kalman filter for transfer alignment on a moving base. Lim and Lyou [13] designed a transfer alignment error compensation method based on H∞ filter to reduce the alignment errors caused by time delay and ship body flexure. In [20] an H∞ optimization method was used to conduct transfer alignment, which estimated the misalignment angle and improved the standard Kalman filter performance when the system noises were not fully known. Gao et al. [19] devised a robust adaptive filter, which lowered the requirement for precise kinematic and observation models for transfer alignment compared with a traditional Kalman filter. By adding robust elements in the filtering process, the influence caused by systematic model errors and the disturbances of dynamic external environment can be effectively reduced [20,22,23,24,25,26,27,28,29,30,31,32].

In this paper, the transfer alignment system model is established in detail and the measurement of velocity matching is adopted. Then the time delay is analyzed and an H∞ filtering method with delay compensation is presented. In order to improve the transfer alignment accuracy of H∞ filtering method with delay compensation, an adaptive H∞ filtering method with delay compensation is devised. The new filtering method can adaptively adjust the robustness factor according to the dynamic external environment. Vehicle transfer alignment experiments verified the effectiveness of the proposed method and the alignment accuracy is dramatically improved.

The rest of this paper is organized as follows: the transfer alignment model containing SINS error dynamics model and measurement model is established in Section 2. In Section 3, an adaptive H∞ filter method with delay compensation for SINS’s transfer alignment is designed and analyzed in detail. In Section 4, vehicle transfer alignment experiment is carried out to verify the effectiveness of the proposed method. Finally, conclusions are drawn in Section 5.

## 2. Transfer Alignment Model

### 2.1. SINS Error Dynamics Model

In this paper, “East-North-Up (ENU)” is defined as the navigation frame, and “Right-Forward-Up” is defined as the body frame. The SINS processes the output of inertial components based on the mathematical equations to obtain the navigation data [2]. Although the various types of system mathematical equations are not identical, they all come from the same equation, the specific force equation. Specific force equation describes the analytical relationship between the accelerometer output and the carrier’s velocity [13,16], it can be expressed in the navigation frame *n* as follows:

(1) $${\dot{V}}^{n}=f^{n}-(2\omega_{ie}^{n}+\omega_{en}^{n})\times V^{n}+g^{n}$$

where $V^{n}$ is the carrier’s velocity; ${\dot{V}}^{n}$ is the derivative of carrier’s velocity; $f^{n}$ is the specific force, which can be measured by the accelerometer; $\omega_{ie}^{n}$ is the Earth’s rotation rate; $\omega_{en}^{n}$ is the angular rate of the earth frame relative to the navigation frame; $(2\omega_{ie}^{n}+\omega_{en}^{n})\times V^{n}$ is the harmful acceleration caused by the Earth rotation and the rotation caused by the carrier’s translational movement; $g^{n}$ is the acceleration of gravity.

The attitude angle error of SINS is influenced by instruction angular velocity and gyro bias. Besides, gyro bias causes the growth of attitude angle error in the opposite direction. The attitude error equation in the navigation frame *n* can be expressed as follows [3,13]:

(2) $$\dot{\phi }=\phi \times \omega_{in}^{n}+\delta \omega_{in}^{n}-\varepsilon^{n}$$

where ϕ is attitude angle error; $\dot{\phi }$ is the derivative of attitude angle error; $\omega_{in}^{n}$ is the angular rate of the inertial frame relative to the navigation frame; $\delta \omega_{in}^{n}$ is the derivative of $\omega_{in}^{n}$; $\varepsilon^{n}$ is the gyro bias.

In system equations, the accelerometer bias and the gyro bias are assumed not to change over time, thus:

(3) $${\dot{\nabla }}^{n}=0$$

(4) $${\dot{\varepsilon }}^{n}=0$$

The model used in Equations (3) and (4) is a model of a random constant which corresponds to a model of an exponentially correlated process when time goes to be endless. In the model of transfer alignment, 10 dimensions’ states are selected to establish the equation of states. The system’s states can be expressed as follows:

(5) $$X(t)={\left[\delta V_{e},\delta V_{n},\phi_{e},\phi_{n},\phi_{u},\nabla_{x},\nabla_{y},\varepsilon_{x},\varepsilon_{y},\varepsilon_{z}\right]}^{T}$$

where *δV_e_* and *δV_n_* are the east velocity error and north velocity error respectively in the navigation frame; *φ_e_*, *φ_n_*, *φ_u_* are the east misalignment angle, north misalignment angle, azimuth misalignment angle respectively in the navigation frame; ∇*_x_*, ∇*_y_* are the right-axis and forward-axis accelerometer bias respectively in the body frame; *ε_x_*, *ε_y_*, *ε_z_* are the right-axis, forward-axis, up-axis gyro bias respectively in the body frame.

Thus, the system’s state equation can be expressed as follows:

(6) $$\dot{X}(t)=F(t)X(t)+W(t)$$

where X(t) is (*n* × 1) state estimate, $\dot{X}(t)$ is (*n* × 1) one step predicted state, variable W(t) is zero mean Gaussian white noise. F(t) is (*n* × *n*) state transition matrix, it can be expressed in concrete formulas as:

(7) $$F(t)=[\begin{array}{l} \begin{array}{cccccccccc} \frac{V_{n}}{R_{M}}tgL & 2\omega_{ie}\sin L+\frac{V_{e}}{R_{N}}tgL & 0 & -f_{u} & f_{n} & T_{11} & T_{12} & 0 & 0 & 0\\ -2\left(\omega_{ie}\sin L+\frac{V_{e}}{R_{N}}tgL\right) & 0 & f_{u} & 0 & -f_{e} & T_{21} & T_{22} & 0 & 0 & 0\\ 0 & -\frac{1}{R_{M}} & 0 & \omega_{ie}\sin L+\frac{V_{e}}{R_{N}}tgL & -\left(\omega_{ie}\cos L+\frac{V_{e}}{R_{N}}\right) & 0 & 0 & -T_{11} & -T_{12} & -T_{13}\\ \frac{1}{R_{N}} & 0 & -\omega_{ie}\sin L-\frac{V_{e}}{R_{N}}tgL & 0 & -\frac{V_{n}}{R_{M}} & 0 & 0 & -T_{21} & -T_{22} & -T_{23}\\ \frac{tgL}{R_{N}} & 0 & \omega_{ie}\cos L+\frac{V_{e}}{R_{N}} & \frac{V_{n}}{R_{M}} & 0 & 0 & 0 & -T_{31} & -T_{32} & -T_{33} \end{array}\\ 0_{5\times 10} \end{array}]$$

where *f_e_*, *f_n_* and *f_u_* are east component, north component and up component of the specific force respectively; *V_e_* and *V_n_* are the east velocity and north velocity respectively; *R_M_* is radius of curvature in meridian and *R_N_* is radius of curvature in prime vertical; *L* is the latitude; *ω_ie_* is the earth rotation rate. The S-SINS attitude matrix is defined as $C_{b}^{n}={\left[T_{ij}\right]}_{3\times 3}$.

### 2.2. Measurement Model

In the model of transfer alignment, velocity differences between M-SINS and S-SINS are adopted as measurement [15]. Therefore, the measurement of velocity matching can be calculated as follows:

(8) $$Y(t)=\left[\begin{array}{l} Y_{E}\\ Y_{N} \end{array}\right]=\left[\begin{array}{c} {\tilde{V}}_{E}^{s}-V_{E}^{m}\\ {\tilde{V}}_{N}^{s}-V_{N}^{m} \end{array}\right]$$

where Y(t) is velocity measurement at time *k*; $Y_{E}$ is east velocity measurement component and $Y_{N}$ is north velocity measurement component; ${\tilde{V}}_{E}^{s}$ and $V_{E}^{m}$ are the S-SINS’s east velocity and M-SINS’s east velocity respectively; ${\tilde{V}}_{N}^{s}$ and $V_{N}^{m}$ are the S-SINS’s north velocity and M-SINS’s north velocity respectively.

The system’s measurement equation can be expressed as follows:

(9) Y(t)=H(t)X(t)+V(t)

where ***V***(*t*) is measurement noise, ***H***(*t*) is measurement matrix and

$$H(t)=\left[\begin{array}{cccccccccc} 1 & 0 & 0 & 0 & 0 & 0 & 0 & 0 & 0 & 0\\ 0 & 1 & 0 & 0 & 0 & 0 & 0 & 0 & 0 & 0 \end{array}\right].$$

## 3. Adaptive H∞ Filtering Method with Delay Compensation

### 3.1. Analysis of Time Delay

**Figure 1:**
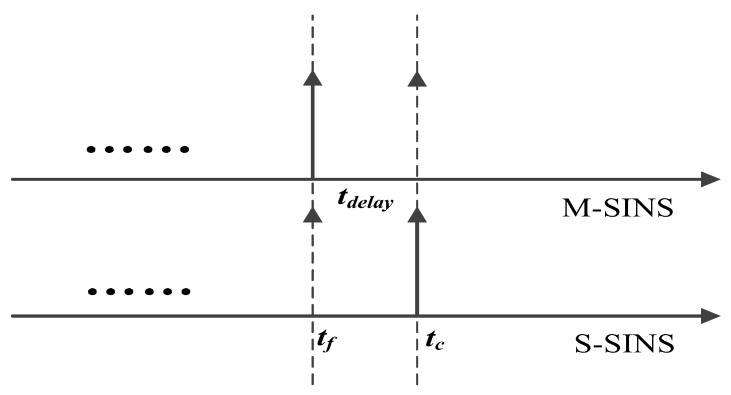
Schematic diagram of time delay.

M-SINS transfers the relevant baseline information to S-SINS during the period of transfer alignment [11]. As shown in Figure 1, ***t**_f_* is the moment when M-SINS begins to send the baseline information, ***t**_c_* is the moment when S-SINS receives the baseline information from M-SINS, and ***t**_delay_* is equal to ***t**_c_*–***t**_f_*, which represents the delay time. S-SINS has time delay problem when receiving the baseline information from M-SINS. The time delay can be caused by the inconsistency of startup time, the existence of transmission delays or the reaction time during the sensors’ processing. The error resulted from time delay reduces the accuracy of baseline information [9]. If we use the unmatched measurement information between M-SINS and S-SINS to conduct filtering, the accuracy of filtering estimation will be affected, which further affects the performance of transfer alignment [10,14]. Therefore, it is necessary to estimate and compensate the delay time to implement the time synchronization between M-SINS and S-SINS.

In this paper, a time delay estimation method based on attitude characteristics is proposed. The time delay will result in attitude error between M-SINS and S-SINS [14]. For a short period of time after the M-SINS and S-SINS begin to work, the impact factors of sensor errors and flexure deformation are small. Therefore, the attitude error is mainly caused by time delay. In the time delay estimation method based on attitude characteristics, the initial attitude data of M-SINS and S-SINS in the period *t*_0_ are recorded. The length of initial attitude data is represented as *N*. Then the range of time delay should be defined according to the condition of the actual system. After that the attitude data of S-SINS should be shifted point by point within the range of time delay. At each of the shift point, the sum of attitude error squares between M-SINS and S-SINS is calculated, it can be expressed as follows:

(10) $$Sum(n)=\sum_{i=n+1}^{N}\left\{{\left[H_{M}(i)-H_{S}(i-n)\right]}^{2}+{\left[P_{M}(i)-P_{S}(i-n)\right]}^{2}+{\left[R_{M}(i)-R_{S}(i-n)\right]}^{2}\right\}$$

where *n* is shift point; *H_M_*, *P_M_*, *R_M_* are heading angle, pitch angle and roll angle of M-SINS respectively; *H_S_*, *P_S_*, *R_S_* are heading angle, pitch angle and roll angle of S-SINS, respectively.

After calculating the sum of attitude errors’ squares at each of the shift point, the minimum values of the sum of attitude errors’ squares can be determined as follows:

(11) $$Sum\left(n_{\min}\right)=\min Sum\left(n\right)$$

where *n*_min_ is the minimum shift point.

And the delay time *τ* of S-SINS can be expressed as follows:

(12) $$\tau =T_{SINS}n_{\min}$$

where *T_SINS_* is the attitude updating cycle of S-SINS.

### 3.2. H∞ Filtering Method with Delay Compensation

#### 3.2.1. H∞ Filtering Theory with Delay Compensation

As shown in Section 2, we consider the following random linear discrete-time system in Krein space [29]:

(13) $$X_{k}=\Phi_{k,k-1}X_{k-1}+W_{k-1}$$

(14) $$Y_{k}=H_{k}X_{k}+V_{k}$$

In general, we need to estimate some arbitrary linear combination of the state, it can be shown as [25]:

(15) $$Z_{k}=L_{k}X_{k}$$

where ***L**_k_* is (*n* × *n*) matrix, ***Z**_k_* is (*n* × 1) measurement matrix. We can use ***Z**_k_* to expand observed state of ***Y**_k_* and the equations below are obtained:

(16) $$X_{k}=\Phi_{k,k-1}X_{k-1}+W_{k-1}$$

(17) $$\left[\begin{array}{l} Y_{k}\\ Z_{k} \end{array}\right]=\left[\begin{array}{l} H_{k}\\ L_{k} \end{array}\right]X_{k}+V_{k}^{\prime }$$

In the above equations ***W**_k_*_−1_ and $V_{k}^{\prime }$ are noise with bounded energy, and we do not make assumptions about their statistical properties. Then *X*_0_, ***W**_k_*_−1_ and $V_{k}^{\prime }$ satisfy the following relationships:

(18) $$\langle \left[\begin{array}{c} X_{0}\\ W_{j-1}\\ V_{j}^{\prime } \end{array}\right],\left[\begin{array}{c} X_{0}\\ W_{k-1}\\ {V^{\prime }}_{k} \end{array}\right]\rangle =[\begin{array}{ccc} P_{0} & 0 & 0\\ 0 & I\delta_{jk} & 0\\ 0 & 0 & R_{\infty k}\delta_{jk} \end{array}]$$

where:

(19) $$R_{\infty k}=\left[\begin{array}{cc} I & 0\\ 0 & -\xi^{2}I \end{array}\right]\delta_{jk}$$

In Equation (19), *δ_jk_* is Kronecker function, i.e., $\delta_{jk}=\left\{ \begin{array}{l} 0\;(k\neq j)\\ 1\;(k=j) \end{array}\right.$; *ξ* is robust factor of H∞ filter.

As an indefinite covariance matrix in H∞ filter, matrix $R_{\infty k}$ is a diagonal matrix. When *ξ* tends to infinity (*ξ*→∞), H∞ filter degenerates to standard Kalman filter. This means that H∞ norm is very big in Kalman filter and the robustness of Kalman filter is bad compared with H∞ filter.

Define:

(20) $${\hat{Z}}_{k}=F_{f}\left(Y_{0},Y_{1},\cdots ,Y_{k}\right)$$

It means the estimation of ***Z**_k_* in the condition of given observation value ***Y**_k_*, the filtering error can be defined as follows:

(21) $$e_{k}={\hat{Z}}_{k}-L_{k}X_{k}$$

Define ***T**_k_*(***F**_f_*) as the transfer function from unknown disturbance $\left\{(X_{0}-{\hat{X}}_{0}),W_{k},V_{k},k\in [0,-\;\infty )\right\}$ to filtering error $\left\{e_{k},k\in [0,-\;\infty )\right\}$, then the H∞ filter problem can be described as follows:

**Definition 1** (Optimal H∞ filter problem)**.** *Find the optimal estimation of H∞ filter to minimize ‖**T**_k_(**F**_f_)‖_∞_, that is [26]:*

(22) $$\xi^{2}=\underset{F_{f}}{\inf}{\left\|T_{k}(F_{f})\right\|}_{\infty }^{2}=\underset{F_{f}}{\inf}\underset{X_{0},W\in h_{2},V\in h_{2}}{\sup}\frac{{\left\|e_{k}\right\|}_{2}^{2}}{{\left\|X_{0}-{\hat{X}}_{0}\right\|}_{P_{0}^{-1}}^{2}+{\left\|W_{k}\right\|}_{2}^{2}+{\left\|{V^{\prime }}_{k}\right\|}_{2}^{2}}$$

In the above equation, *P*_0_ is a positive definite matrix. Symbol “inf” is the infimum of function and symbol “sup” is the supremum of function. Optimal H∞ filter ensures the minimized energy gain of estimation error for all possible input with certain energy. However, the result is too conservative, and the definition of suboptimal H∞ filter problem is given below.

**Definition 2** (Suboptimal H∞ filter problem)**.** *When given a positive number ξ > 0, find suboptimal H∞ estimation to satisfy ‖**T**_k_(**F**_f_)‖_∞_ < ξ, that is [25,29]:*

(23) $$\underset{F_{f}}{\inf}{\left\|T_{k}(F_{f})\right\|}_{\infty }^{2}=\underset{F_{f}}{\inf}\underset{X_{0},W\in h_{2},V\in h_{2}}{\sup}\frac{{\left\|e_{k}\right\|}_{2}^{2}}{{\left\|X_{0}-{\hat{X}}_{0}\right\|}_{P_{0}^{-1}}^{2}+{\left\|W_{k}\right\|}_{2}^{2}+{\left\|V_{k}^{\prime }\right\|}_{2}^{2}}\leq \xi^{2}$$

**Theorem 1** (Suboptimal H∞ filter problem)**.** *When given a positive number ξ > 0, if [**Φ**_k,k−1_**Γ**_k,k−1_] is full rank, then the condition that there is a filter satisfying ‖**T**_k_(**F**_f_)‖_∞_ < ξ is if and only if [23]*

(24) $$P_{k}^{-1}+H_{k}^{T}H_{k}-\xi^{-2}L_{k}^{T}L_{k}>0$$

In the above equation, ***P**_k_* satisfies the recurrent Riccati equation:

(25) $$P_{k}=\Phi_{k,k-1}P_{k-1}\Phi_{k,k-1}^{T}+\Gamma_{k,k-1}\Gamma_{k,k-1}^{T}-\Phi_{k,k-1}P_{k-1}\left[\begin{array}{cc} H_{k}^{T} & L_{k}^{T} \end{array}\right]R_{e,k}^{-1}\left[\begin{array}{c} H_{k}\\ L_{k} \end{array}\right]P_{k-1}\Phi_{k,k-1}^{T}$$

In Equation (25):

(26) $$R_{e,k}=\left[\begin{array}{cc} I & 0\\ 0 & -\xi^{2}I \end{array}\right]+\left[\begin{array}{c} H_{k}\\ L_{k} \end{array}\right]P_{k-1}\left[\begin{array}{cc} H_{k}^{T} & L_{k}^{T} \end{array}\right]$$

Taking into account the effect of time delay, the recursive equations of H∞ filter can be expressed as follows:

(27) $${\hat{X}}_{k-\tau ,k-\tau -1}=\Phi_{k-\tau ,k-\tau -1}{\hat{X}}_{k-\tau -1}$$

(28) $$R_{e,k-\tau -1}=\left[\begin{array}{cc} I & 0\\ 0 & -\xi^{2}I \end{array}\right]+\left[\begin{array}{c} H_{k-\tau -1}\\ L_{k-\tau -1} \end{array}\right]P_{k-\tau -1}\left[\begin{array}{cc} H_{k-\tau -1}^{T} & L_{k-\tau -1}^{T} \end{array}\right]$$

(29) $$P_{k-\tau }=\Phi_{k-\tau ,k-\tau -1}P_{k-\tau -1}\Phi_{k-\tau ,k-\tau -1}^{T}-\Phi_{k-\tau ,k-\tau -1}P_{k-\tau -1}\left[\begin{array}{cc} H_{k-\tau -1}^{T} & L_{k-\tau -1}^{T} \end{array}\right]R_{e,k-\tau -1}^{-1}\left[\begin{array}{c} H_{k-\tau -1}\\ L_{k-\tau -1} \end{array}\right]P_{k-\tau -1}\Phi_{k-\tau ,k-\tau -1}^{T}+\Gamma_{k-\tau -1}\Gamma_{k-\tau -1}^{T}$$

(30) $$K_{k-\tau }=P_{k-\tau }H_{k-\tau }^{T}{\left(I+H_{k-\tau }P_{k-\tau }H_{k-\tau }^{T}\right)}^{-1}$$

(31) $${\hat{X}}_{k-\tau }={\hat{X}}_{k-\tau ,k-\tau -1}+K_{k-\tau }\left(Y_{k-\tau }-H_{k-\tau }{\hat{X}}_{k-\tau ,k-\tau -1}\right)$$

According to the above recursive equations, the estimation result is the system error state ${\hat{X}}_{T_{P}-\tau }$ at the moment *T_P−_τ*, where *T_P_* is the current moment, *τ* is the delay time of the system. So when the filtering estimation process is completed, the system error state should be converted to the current moment *T_P_*. Define *T_HF-D_* as the update cycle of H∞ Filter with delay compensation, the system error state at the current moment can be expressed as follows:

(32) $${\hat{X}}_{T_{P}}=e^{F(T_{P}-[\tau /T_{\mathrm{SIN}S}]T_{\mathrm{SIN}S})T_{HF-D}}{\hat{X}}_{T_{P}-\tau }$$

#### 3.2.2. Robust Mechanism Analysis of H∞ Filtering Theory

Next the detailed analysis on the robust mechanism of H∞ filtering theory is presented. By organizing *P_k−τ_* in Equation (28), the following equation can be obtained:

(33) $$P_{k}=\Phi_{k,k-1}\left(I-P_{k-1}\left[\begin{array}{cc} H_{k}^{T} & L_{k}^{T} \end{array}\right]{\left(\left[\begin{array}{cc} I & 0\\ 0 & -\xi^{2}I \end{array}\right]+\left[\begin{array}{c} H_{k}\\ L_{k} \end{array}\right]P_{k-1}\left[\begin{array}{cc} H_{k}^{T} & L_{k}^{T} \end{array}\right]\right)}^{-1}[\begin{array}{c} H_{k}\\ L_{k} \end{array}]\right)P_{k-1}\Phi_{k,k-1}^{T}+\Gamma_{k,k-1}\Gamma_{k,k-1}^{T}$$

Define:

(34) $$J_{\infty ,k}=P_{k-1}\left[\begin{array}{cc} H_{k}^{T} & L_{k}^{T} \end{array}\right]{\left(\left[\begin{array}{cc} I & 0\\ 0 & -\xi^{2}I \end{array}\right]+\left[\begin{array}{c} H_{k}\\ L_{k} \end{array}\right]P_{k-1}\left[\begin{array}{cc} H_{k}^{T} & L_{k}^{T} \end{array}\right]\right)}^{-1}$$

Then:

(35) $$P_{k}=\Phi_{k,k-1}\left(I-J_{\infty ,k}[\begin{array}{c} H_{k}\\ L_{k} \end{array}]\right)P_{k-1}\Phi_{k,k-1}^{T}+\Gamma_{k,k-1}\Gamma_{k,k-1}^{T}$$

Next $J_{\infty ,k}$ in Equation (34) is simplified as follows:

(36) $$J_{\infty ,k}=P_{k-1}\left[\begin{array}{cc} H_{k}^{T} & L_{k}^{T} \end{array}\right]{\left(\left[\begin{array}{c} H_{k}\\ L_{k} \end{array}\right]P_{k-1}\left[\begin{array}{cc} H_{k}^{T} & L_{k}^{T} \end{array}\right]+\left[\begin{array}{cc} I & 0\\ 0 & -\xi^{2}I \end{array}\right]\right)}^{-1}\;=\left[\begin{array}{cc} P_{k-1}H_{k}^{T} & P_{k-1}L_{k}^{T} \end{array}\right]{\left[\begin{array}{cc} I+H_{k}P_{k-1}H_{k}^{T} & H_{k}P_{k-1}L_{k}^{T}\\ L_{k}P_{k-1}H_{k}^{T} & -\xi^{2}I+L_{k}P_{k-1}L_{k}^{T} \end{array}\right]}^{-1}$$

According to the inverse theorem of partitioned matrix, we define:

(37) $$\begin{array}{l} \alpha_{11}=I+H_{k}P_{k-1}H_{k}^{T}\\ \alpha_{12}=H_{k}P_{k-1}L_{k}^{T}\\ \alpha_{21}=L_{k}P_{k-1}H_{k}^{T}\\ \alpha_{22}=-\xi^{2}I+L_{k}P_{k-1}L_{k}^{T} \end{array}$$

Let:

(38) $$\alpha^{-1}=\left[\begin{array}{cc} \beta_{11} & \beta_{12}\\ \beta_{21} & \beta_{22} \end{array}\right]$$

where $\beta_{\;22}={\left(\alpha_{22}-\alpha_{21}\alpha_{11}^{-1}\alpha_{12}\right)}^{-1}$, $\beta_{\;12}=-\alpha_{11}^{-1}\alpha_{12}\beta_{22}$, $\beta_{\;21}=-\beta_{22}\alpha_{21}\alpha_{11}^{-1}$, $\beta_{\;11}=\alpha_{11}^{-1}-\beta_{12}\alpha_{21}\alpha_{11}^{-1}$.

Then:

(39) $$J_{\infty ,k}=\left[\begin{array}{cc} P_{k-1}H_{k}^{T} & P_{k-1}L_{k}^{T} \end{array}\right]\left[\begin{array}{cc} \beta_{11} & \beta_{12}\\ \beta_{21} & \beta_{22} \end{array}\right]=\left[\begin{array}{cc} P_{k-1}H_{k}^{T}\beta_{11}+P_{k-1}L_{k}^{T}\beta_{21} & P_{k-1}H_{k}^{T}\beta_{12}+P_{k-1}L_{k}^{T}\beta_{22} \end{array}\right]$$

Here we define ***J***_∞,*k*_ = [***J**_P_* ***J**_Q_*] and bring $\alpha_{11}$, $\alpha_{12}$, $\alpha_{21}$ and $\alpha_{22}$ into Equation (39). Firstly, ***J**_P_* can be simplified as follows:

(40) $$\begin{array}{ll} J_{P} & =P_{k-1}H_{k}^{T}\beta_{11}+P_{k-1}L_{k}^{T}\beta_{21}\\ & =P_{k-1}H_{k}^{T}\left(\alpha_{11}^{-1}-\beta_{12}\alpha_{21}\alpha_{11}^{-1}\right)+P_{k-1}L_{k}^{T}\left(-\beta_{22}\alpha_{21}\alpha_{11}^{-1}\right)\\ & =P_{k-1}H_{k}^{T}{\left(I+H_{k}P_{k-1}H_{k}^{T}\right)}^{-1}+P_{k-1}\left(H_{k}^{T}\alpha_{11}^{-1}\alpha_{12}-L_{k}^{T}\right){\left(\alpha_{22}-\alpha_{21}\alpha_{11}^{-1}\alpha_{12}\right)}^{-1}L_{k}P_{k-1}H_{k}^{T}{\left(I+H_{k}P_{k-1}H_{k}^{T}\right)}^{-1}. \end{array}$$

Define $J_{S,k}=P_{k-1}H_{k}^{T}{\left(I+H_{k}P_{k-1}H_{k}^{T}\right)}^{-1}$, then Equation (40) can be simplified as:

(41) $$\begin{array}{ll} J_{P} & =J_{S,k}+P_{k-1}\left(H_{k}^{T}\alpha_{11}^{-1}\alpha_{12}-L_{k}^{T}\right){\left(\alpha_{22}-\alpha_{21}\alpha_{11}^{-1}\alpha_{12}\right)}^{-1}L_{k}J_{S,k}\\ & =\left[I+P_{k-1}\left(H_{k}^{T}\alpha_{11}^{-1}\alpha_{12}-L_{k}^{T}\right){\left(\alpha_{22}-\alpha_{21}\alpha_{11}^{-1}\alpha_{12}\right)}^{-1}L_{k}\right]J_{S,k}\\ & =\left\{I+\left(J_{S,k}H_{k}-I\right)P_{k-1}L_{k}^{T}{\left[-\xi^{2}I+L_{k}\left(I-J_{S,k}H_{k}\right)P_{k-1}L_{k}^{T}\right]}^{-1}L_{k}\right\}J_{S,k} \end{array}$$

Define ${ϒ}_{k}=\left(I-J_{S,k}H_{k}\right)P_{k-1}$, then Equation (41) can be described as:

(42) $$J_{P}=\left[I-{ϒ}_{k}L_{k}^{T}{\left(-\xi^{2}I+L_{k}{ϒ}_{k}L_{k}^{T}\right)}^{-1}L_{k}\right]J_{S,k}$$

and we define $G_{k}={ϒ}_{k}L_{k}^{T}{\left(-\xi^{2}I+L_{k}{ϒ}_{k}L_{k}^{T}\right)}^{-1}L_{k}$, thus Equation (42) can be described as:

(43) $$J_{P}=\left(I-G_{k}\right)J_{S,k}$$

Secondly, we can simplify ***J**_Q_* in the similar way as follows:

(44) $$\begin{array}{ll} J_{Q} & =P_{k-1}H_{k}^{T}\beta_{12}+P_{k-1}L_{k}^{T}\beta_{22}\\ & =\left[I-P_{k-1}H_{k}^{T}{\left(I+H_{k}P_{k-1}H_{k}^{T}\right)}^{-1}H_{k}\right]P_{k-1}L_{k}^{T}{\left[-\xi^{2}I+L_{k}\left(I-P_{k-1}H_{k}^{T}{\left(I+H_{k}P_{k-1}H_{k}^{T}\right)}^{-1}H_{k}\right)P_{k-1}L_{k}^{T}\right]}^{-1}\\ & ={ϒ}_{k}L_{k}^{T}{\left(-\xi^{2}I+L_{k}{ϒ}_{k}L_{k}^{T}\right)}^{-1} \end{array}$$

After carrying out the simplification operation above, Equations (43) and (44) are brought into ***J***_∞,*k*_ = [***J**_P_* ***J***_Q_], then Equation (35) can be represented as follows:

(45) $$\begin{array}{ll} P_{k} & =\Phi_{k,k-1}\left(I-\left[\begin{array}{cc} \left(I-G_{k}\right)J_{S,k} & {ϒ}_{k}L_{k}^{T}{\left(-\xi^{2}I+L_{k}{ϒ}_{k}L_{k}^{T}\right)}^{-1} \end{array}\right][\begin{array}{c} H_{k}\\ L_{k} \end{array}]\right)P_{k-1}\Phi_{k,k-1}^{T}+\Gamma_{k,k-1}\Gamma_{k,k-1}^{T}\\ & =\Phi_{k,k-1}P_{k-1}\Phi_{k,k-1}^{T}-\Phi_{k,k-1}\left[\left(I-G_{k}\right)J_{S,k}H_{k}+G_{k}\right]P_{k-1}\Phi_{k,k-1}^{T}+\Gamma_{k,k-1}\Gamma_{k,k-1}^{T} \end{array}$$

Taking the effect of time delay into consideration, the H∞ filter recurrence equations which are simplified by decomposition of matrix can be expressed as follows:

(46) $${\hat{X}}_{k-\tau ,k-\tau -1}=\Phi_{k-\tau ,k-\tau -1}{\hat{X}}_{k-\tau -1}$$

(47) $$J_{S,k-\tau }=P_{k-\tau -1}H_{k-\tau }^{T}{\left(I+H_{k-\tau }P_{k-\tau -1}H_{k-\tau }^{T}\right)}^{-1}$$

(48) $${ϒ}_{k-\tau }=\left(I-J_{S,k-\tau }H_{k-\tau }\right)P_{k-\tau -1}$$

(49) $$G_{k-\tau }={ϒ}_{k-\tau }L_{k-\tau }^{T}{\left(-\xi^{2}I+L_{k-\tau }{ϒ}_{k-\tau }L_{k-\tau }^{T}\right)}^{-1}L_{k-\tau }$$

(50) $$P_{k-\tau }=\Phi_{k-\tau ,k-\tau -1}P_{k-\tau -1}\Phi_{k-\tau ,k-\tau -1}^{T}-\Phi_{k-\tau ,k-\tau -1}\left[\left(I-G_{k-\tau }\right)J_{S,k-\tau }H_{k-\tau }+G_{k-\tau }\right]P_{k-\tau -1}\Phi_{k-\tau ,k-\tau -1}^{T}+\Gamma_{k-\tau ,k-\tau -1}\Gamma_{k-\tau ,k-\tau -1}^{T}$$

(51) $$J_{k-\tau }=P_{k-\tau }H_{k-\tau }^{T}{\left(H_{k-\tau }P_{k-\tau }H_{k-\tau }^{T}+I\right)}^{-1}$$

(52) $${\hat{X}}_{k-\tau }={\hat{X}}_{k-\tau ,k-\tau -1}+J_{k-\tau }\left(Y_{k-\tau }-H_{k-\tau }{\hat{X}}_{k-\tau ,k-\tau -1}\right)$$

As the estimation result of the above recursive equations is the system error state ${\hat{X}}_{T_{P}-\tau }$ at the moment *T_P_−τ*. The system error state should be converted to the current moment *T_P_* when the filtering estimation process is completed, and it can be expressed as follows:

(53) $${\hat{X}}_{T_{P}}=e^{F(T_{P}-[\tau /T_{\mathrm{SIN}S}]T_{\mathrm{SIN}S})T_{HF-D}}{\hat{X}}_{T_{P}-\tau }$$

### 3.3. Adaptive H∞ Filtering Method with Delay Compensation

In the H∞ filter with delay compensation, the robustness factor *ξ* plays an important role in filtering accuracy, robustness and stability [24]. When the value of the robustness factor *ξ* is too small, the system has better robustness but the filtering accuracy of the system is too low. When the value of the robustness factor *ξ* is too big, the system has worse stability and even leads to divergence. Therefore, the value of the robustness factor *ξ* has a direct effect on the performance of H∞ filter with delay compensation. In most cases, the value of the robustness factor *ξ* needs to be debugged over and over again before using H∞ filter with delay compensation, and eventually the robustness factor *ξ* of the H∞ filter with delay compensation is often determined according to actual engineering experience. This takes a lot of time and the selected value of *ξ* is always not optimal. Moreover, it cannot be guaranteed that when the filtering estimation error is small, the system also has good robustness at the same time. If there exist large disturbances in the transfer alignment system for some moment, the filter may be unable to improve the robustness because of the fixed robustness factor *ξ,* so the filtering estimation error will increase under the uncertain external environment. To solve the problem, an adaptive optimization method of the robustness factor *ξ* is proposed in this paper to improve the performance of H∞ filter with delay compensation.

During the period of transfer alignment, when the H∞ filter with delay compensation diverges, the error covariance matrix is unbounded and the real estimation error is bigger than the theoretical estimation error [19]. Therefore, we can evaluate the performance of H∞ filter with delay compensation by using filter innovation $\eta_{k-\tau }$. It is well known that filter innovation $\eta_{k-\tau }$ can be expressed as follows:

(54) $$\eta_{k-\tau }=Y_{k-\tau }-H_{k-\tau }{\hat{X}}_{k-\tau /k-\tau -1}$$

Based on Equation (54), the quadratic sum of innovation sequence can be expressed as $\eta_{k-\tau }^{T}\eta_{k-\tau }$, which reflects the real estimation error of filtering [21]. The theoretical prediction error can be described by the innovation sequence’s variance $E\;[\eta_{k-\tau }^{T}\eta_{k-\tau }]$. Under ideal conditions, the filter innovation is zero mean Gaussian white noise sequence, but in real systems, both the changes of system model and the anomaly of observed values can cause the change on statistical properties of innovation sequence [24]. When the value of $\eta_{k-\tau }^{T}\eta_{k-\tau }$ is too big, it means that the performance of the filter degrades, even with the possibility of divergence [21]. Thus the robustness of the adaptive H∞ filter with delay compensation is of great importance at the moment, and the value of the robustness factor *ξ* should be reduced to compensate the large error which is generated by $\eta_{k-\tau }^{T}\eta_{k-\tau }$. When the value of $\eta_{k-\tau }^{T}\eta_{k-\tau }$ is too small, it means that the transfer alignment system suffers fewer external disturbances, so the value of the robustness factor *ξ* can be increased to improve the filtering accuracy, and the robustness of the filter should be reduced properly at that moment. During the whole process of transfer alignment, the robustness factor *ξ* of the adaptive H∞ filter with delay compensation varies inversely with respect to $\eta_{k-\tau }^{T}\eta_{k-\tau }$.

**Theorem** **2.** *Assume that **P** and **Q** are two n-order Hermite matrices, **P** > 0, **Q** ≥ 0, then **P** > **Q** is equal to* $\lambda_{\max}(QP^{-1})\;<\;1$ *[33]. Here λ_max_(**P**) represents the maximum eigenvalue of matrix **P**.*

According to the Theorem 2, we can transform the conditional Equation (24) of H∞ filter with delay compensation to the following form:

(55) $$\xi^{2}>\lambda_{\max}(L_{k-\tau }^{T}L_{k-\tau }{\left(P_{k-\tau }^{-1}+H_{k-\tau }^{T}H_{k-\tau }\right)}^{-1})$$

and the value of *ξ* can be expressed as follows:

(56) $$\xi =(1+\upsilon )\cdot {\left[\lambda_{\max}(L_{k-\tau }^{T}L_{k-\tau }{\left(P_{k-\tau }^{-1}+H_{k-\tau }^{T}H_{k-\tau }\right)}^{-1})\right]}^{1/2}$$

In the above equation, coefficient *υ* > 0. Because the value of *ξ* is inversely proportional to $\eta_{k-\tau }^{T}\eta_{k-\tau }$, we can make *υ* and $\eta_{k-\tau }^{T}\eta_{k-\tau }$ satisfy the following relationship:

(57) $$\upsilon =\frac{\kappa }{\sqrt{\frac{\eta_{k-\tau }^{T}\eta_{k-\tau }}{N}}}$$

where *κ* is correlation coefficient and *κ* > 0, the value of *κ* can be determined by the experiment of the real system. *N* is the dimension of the filtering states. For a specific real system, once the values of *κ* and *N* are determined, respectively, they will not change any more. In this case, the value of coefficient *υ* is only related to filter innovation during the process of transfer alignment. When filter innovation increases, the value of coefficient *υ* decreases, so the robust factor *ξ* of adaptive H∞ filter with delay compensation will be decreased to improve the robustness of the transfer alignment system. On the contrary, when filter innovation decreases, the value of coefficient *ν* increases, so the robustness factor *ξ* of the adaptive H∞ filter with delay compensation will be increased to improve the estimation accuracy of transfer alignment system. Therefore, the relationship between the robustness factor *ξ* of the adaptive H∞ filter with delay compensation and $\eta_{k-\tau }^{T}\eta_{k-\tau }$ can be established, and the value of the robustness factor *ξ* will change dynamically as the filter innovation $\eta_{k-\tau }$ changes.

By comparing the adaptive H∞ filter with delay compensation and the H∞ filter with delay compensation, it can be seen that both methods are able to satisfy Equation (24). However, the robustness factor *ξ* of the adaptive H∞ filter with delay compensation is dynamically adjusted according to the value of filter innovation $\eta_{k-\tau }$. Compared with the H∞ filter with delay compensation, the adaptive H∞ filter with delay compensation can adjust the robustness factor *ξ* adaptively with the change of the real system environment. As a result, the filtering estimation accuracy and robustness of the transfer alignment system can be optimized by using the proposed filtering method.

## 4. Experimental Results and Discussions

### 4.1. Experimental Settings

**Figure 2:**
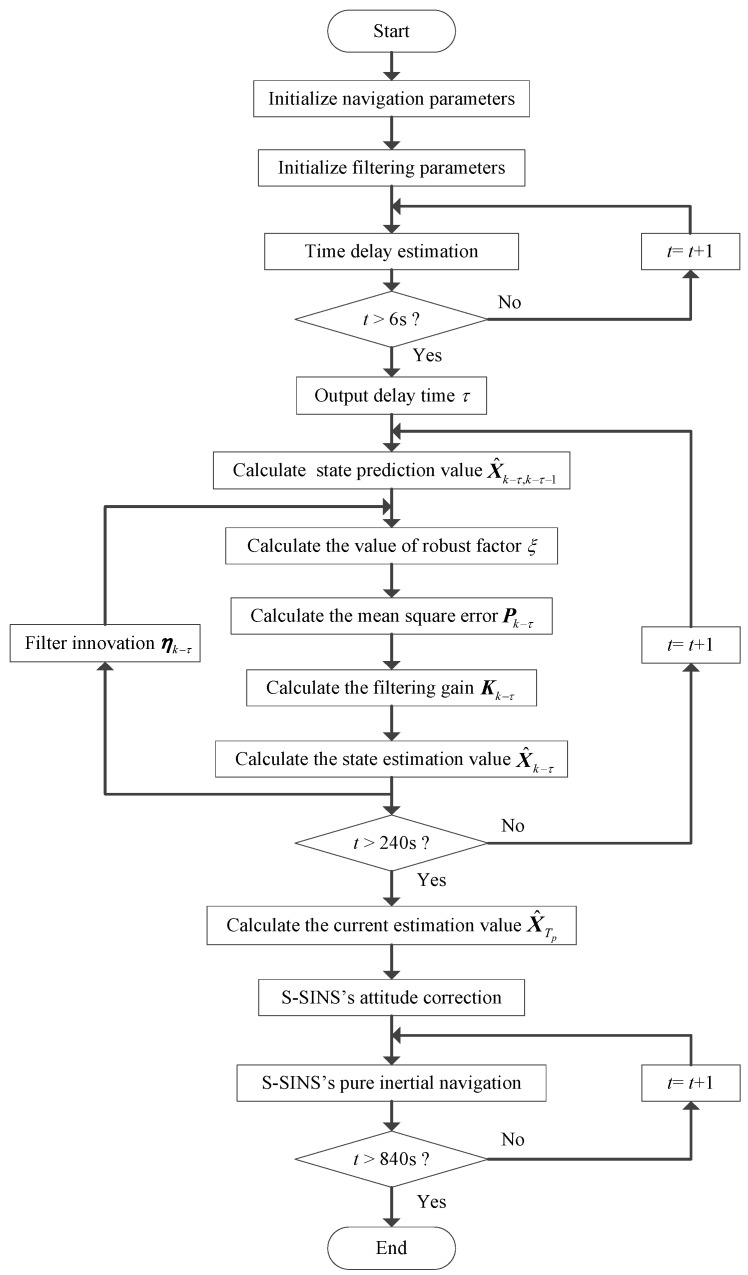
Flow chart of transfer alignment based on adaptive H∞ filtering method with delay compensation.

In order to verify the adaptive H∞ filtering method with delay compensation, a vehicle transfer alignment experiment in a real system is conducted. The real vehicle experiment is carried out in Beijing in a wide-open area. The specific location of this experiment is determined as east longitude 116° and north latitude 39°. The transfer alignment system contains M-SINS, S-SINS and navigation computer, which are connected by communication lines. The position of both M-SINS and S-SINS is kept strictly fixed in the experimental vehicle. The update frequencies for M-SINS and S-SINS are all set as 200 Hz. The strapdown algorithm update cycle is 5 ms. The three-axis gyro biases of S-SINS are 0.6 °/h, and the three-axis accelerometer biases of S-SINS are 0.2 mg. The reference baseline information is provided by M-SINS in the process of transfer alignment, and the sensor parameters of M-SINS are far better than those of S-SINS. The flow chart of transfer alignment based on adaptive H∞ filtering method with delay compensation is presented is shown in Figure 2.

**Figure 3:**
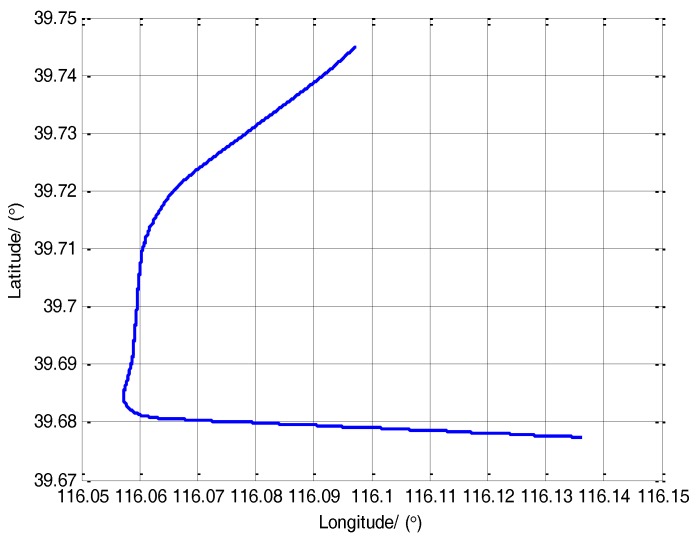
The trajectory of vehicle experiment.

**Figure 4:**
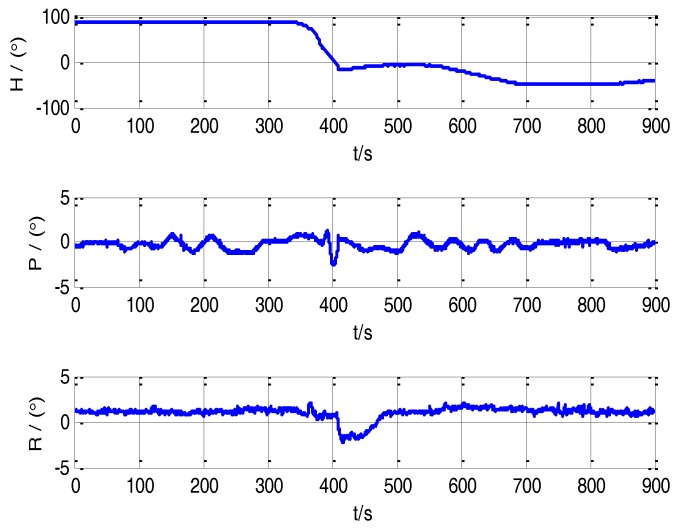
The attitude curve of M-SINS.

Firstly, the transfer alignment system takes 6 s to estimate the time delay. After the time delay estimation, the system takes 234 s to conduct the transfer alignment on a moving base. Then the S-SINS of transfer alignment system begins 600 s’ pure inertial navigation. In the whole process, the navigation computer records the output data of M-SINS and S-SINS which is transmitted through the communication lines in real time. The movement trajectory of the vehicle transfer alignment experiment is presented in Figure 3. During the period of time delay estimation, transfer alignment and pure inertial navigation, the M-SINS’s attitude curves of heading, pitch and roll are as shown in Figure 4.

**Figure 5:**
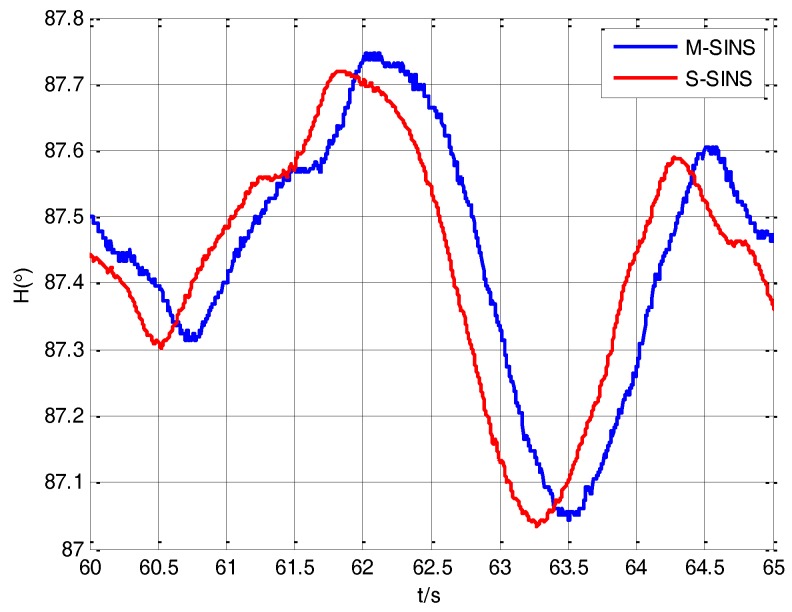
The heading angles’ curves of M-SINS and S-SINS.

**Figure 6:**
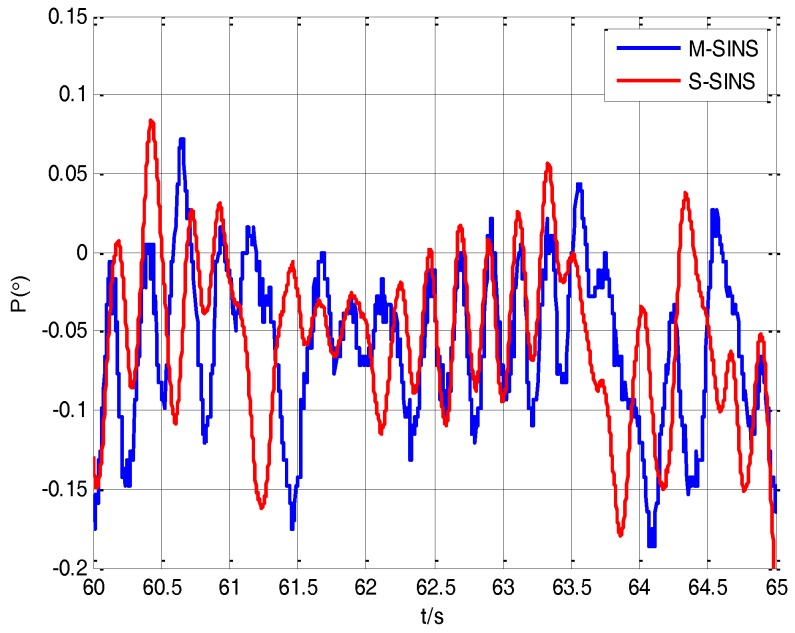
The pitching angles’ curves of M-SINS and S-SINS.

**Figure 7:**
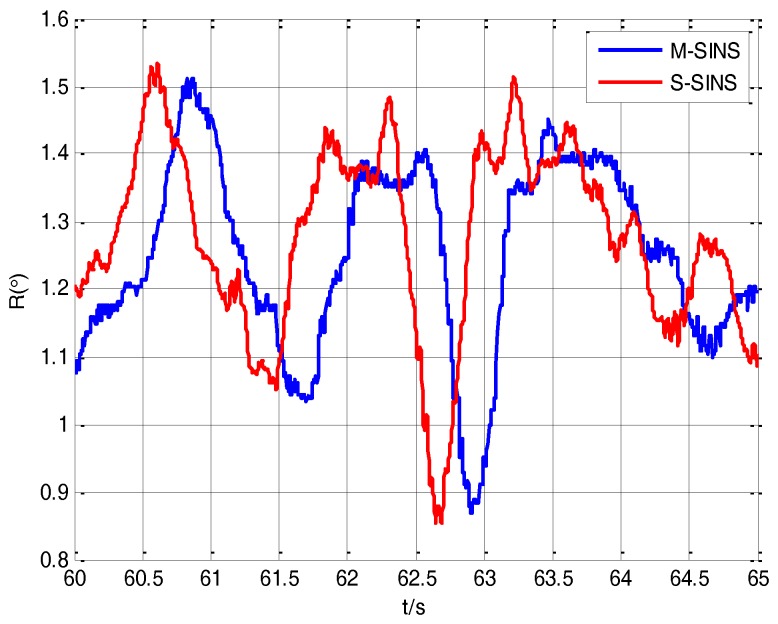
The rolling angles’ curves of M-SINS and S-SINS.

Due to the existence of the time delay between M-SINS and S-SINS, it takes a time delay *τ* to transmit the M-SINS’s baseline information to the S-SINS. Thus at the same moment the S-SINS’s attitudes of heading, pitch and roll are ahead of M-SINS’s attitudes in the navigation computer. The attitude curves of M-SINS and S-SINS from 60 s to 65 s in the period of transfer alignment experiment are presented in Figure 5, Figure 6 and Figure 7. Because the S-SINS just starts to work, the influence of S-SINS’s gyro bias and S-SINS’s accelerometer bias is not very big. The attitudes calculated by the S-SINS look similar to the M-SINS’s attitudes. As time goes on, however, the influence of S-SINS’s sensor parameters become more and more significant.

It can be seen from the figures that during the transfer alignment experiment, the S-SINS’s attitudes of heading, pitch and roll are approximately 0.24 s ahead of M-SINS’s attitudes, which have a negative effect on the transfer alignment accuracy. Therefore, the time delay needs to be effectively estimated and compensated during the transfer alignment process.

### 4.2. Experimental Results and Discussions

In the vehicle transfer alignment experiment, the robustness factor *ξ* of H∞ filter is largely related to the transfer alignment accuracy. The different values of the robustness factor *ξ* can produce different alignment results. It is known that the more accurate the transfer alignment is, the higher the navigation accuracy is, so the different values of the robustness factor *ξ* can result in different navigation accuracy of the S-SINS.

**Figure 8:**
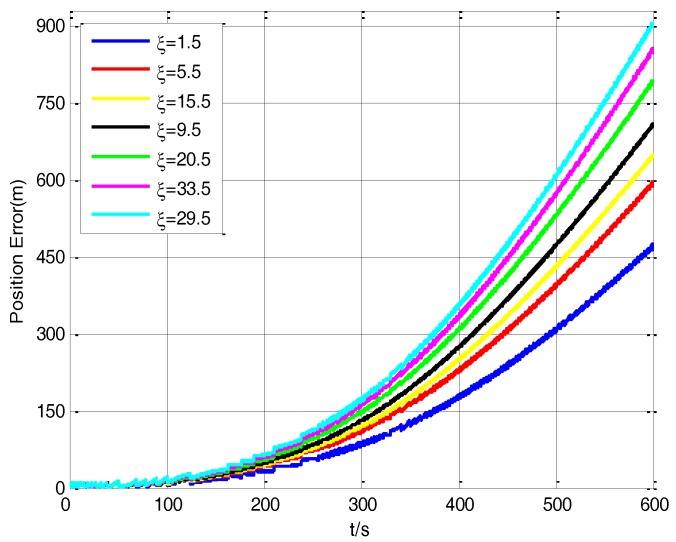
The influence of robustness factors *ξ* on navigation accuracy.

Figure 8 shows the influence of the robustness factors *ξ* on the navigation accuracy of the vehicle experiment. It can be seen that when the robustness factor *ξ* is 1.5, the position error is about 461 m. But when the robustness factor *ξ* is 29.5, the position error is about 905 m. Different robustness factor *ξ* values can lead to big differences of the position errors in the process of vehicle experiment. Therefore, the selected value of the robustness factor *ξ* is important for the H∞ filter performance.

**Figure 9:**
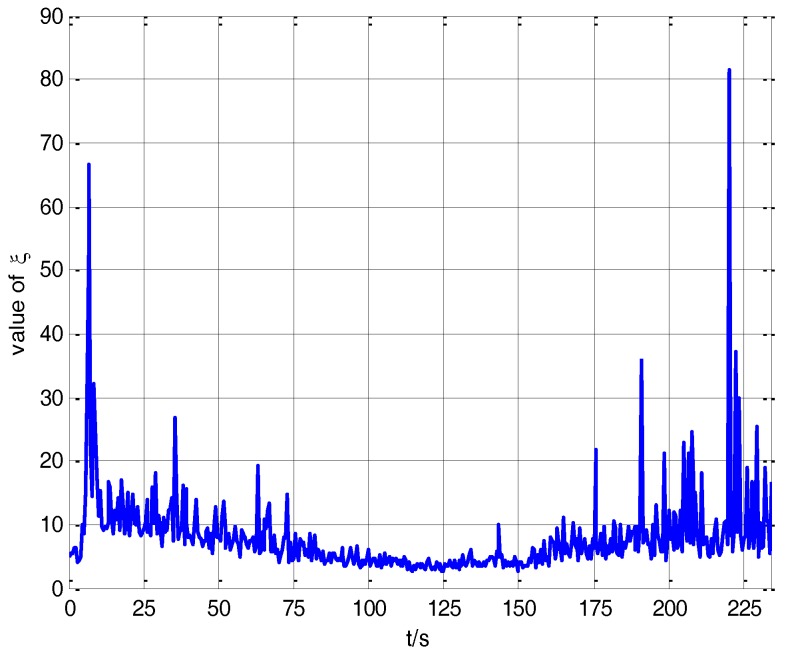
The value of *ξ* in the adaptive H∞ filtering method.

If the value of the robustness factor *ξ* is adjusted dynamically in the filtering process according to the filter innovation, the transfer alignment accuracy can be improved dramatically. Thus an adaptive H∞ filtering method with delay compensation is proposed in this paper, the value of the robustness factor *ξ* in the adaptive H∞ filter can be adjusted dynamically according to the external environment. Figure 9 shows the value of *ξ* in the adaptive H∞ filtering method with delay compensation in the vehicle transfer alignment experiment.

The curve in Figure 9 indicates that during the process of transfer alignment, the robustness factor *ξ* changes adaptively with the real system environment. When the real system environment changes dramatically, the filter innovation increases, and the robustness factor *ξ* will be decreased to improve the robustness of the real system. When the real system environment changes slowly, the filter innovation decreases, and the robustness factor *ξ* will be increased to improve the estimation accuracy of the real system. Therefore, the robustness factor *ξ* is adjusted dynamically with the change of real system environment, which can provide the optimal estimation for the transfer alignment experiment.

**Figure 10:**
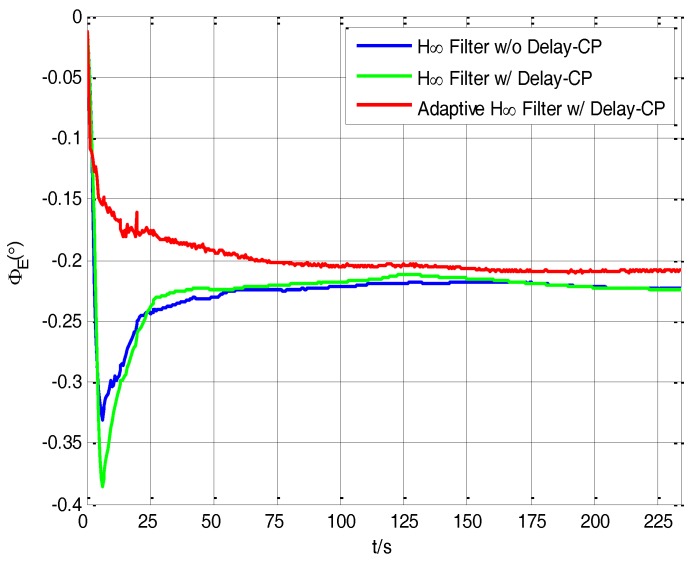
The estimation curves of east misalignment angle.

With the purpose of comparing the performance of different filtering methods, the H∞ filter without delay compensation, the H∞ filter with delay compensation and the adaptive H∞ filter with delay compensation are respectively used in the process of transfer alignment experiment. The robust factor *ξ* of H∞ filter is set as 4.36, which is the optimal robust factor of H∞ filter in this vehicle transfer alignment experiment. The estimation curves of east misalignment angle, north misalignment angle and azimuth misalignment angle using the mentioned filtering algorithms are presented in Figure 8, Figure 9 and Figure 10.

In the figures, the blue lines represent the estimation curves of the H∞ filter without delay compensation (“w/” denotes “with”, “w/o” denotes “without”, ”CP” denotes “compensation”), the green lines represent the estimation curves of the H∞ filter with delay compensation, and the red lines represent the estimation curves of the adaptive H∞ filter with delay compensation. In Figure 10, the estimation curves of *φ_E_* converge to steady values after 175 s’ transfer alignment experiment. But it can be seen that by using the adaptive H∞ filtering method with delay compensation, the estimation value of *φ_E_* is more steady after 175 s than using the H∞ filtering method without delay compensation and the H∞ filtering method with delay compensation.

**Figure 11:**
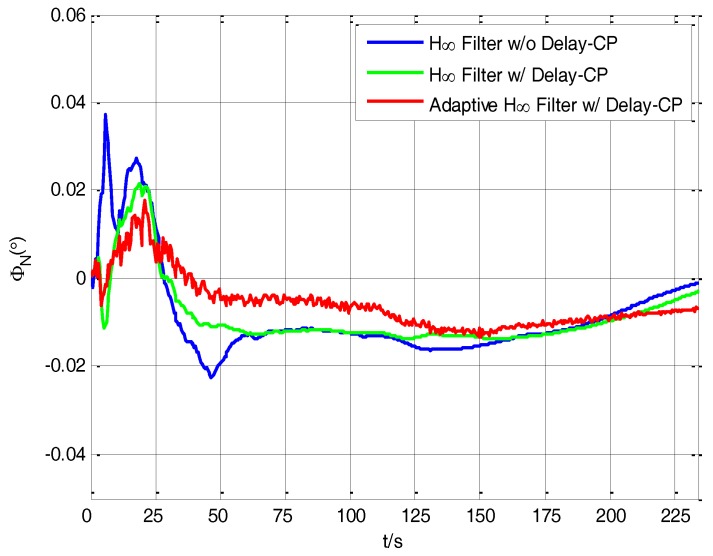
The estimation curves of north misalignment angle.

It can be seen from Figure 11 that by using the H∞ filtering method without delay compensation, the estimation value of *φ_N_* fluctuates dramatically before 50 s, and the estimation value of *φ_N_* still has fluctuation from 50 s to 234 s. Comparatively, the estimation value of *φ_N_* by using the H∞ filtering method with delay compensation is more steady than the method without delay compensation and the fluctuation is effectively reduced. Compared with the two filtering methods above, the estimation value of *φ_N_* has less fluctuation when using adaptive H∞ filtering method with delay compensation. After 175 s, the estimation curve is more steady compared with the other two methods and it can provide more accurate north misalignment angle for S-SINS’s transfer alignment.

**Figure 12:**
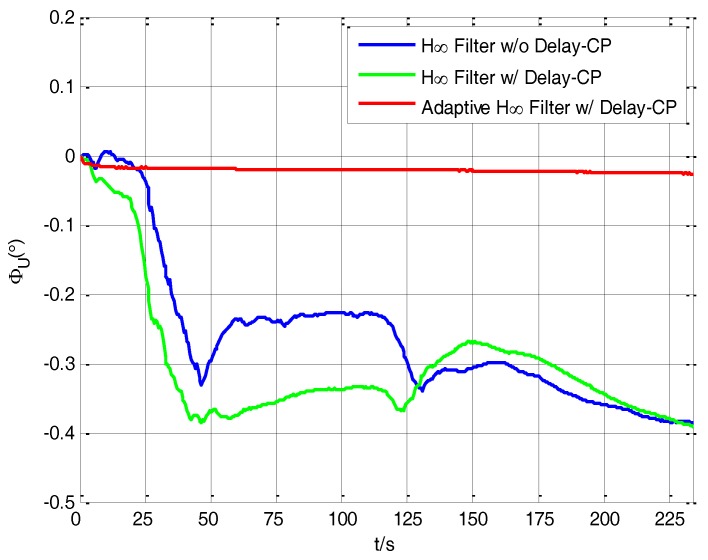
The estimation curves of azimuth misalignment angle.

As shown in Figure 12, the estimation values of *φ_U_* have a relatively large fluctuation during the process of transfer alignment when using the H∞ filtering method without delay compensation and the H∞ filtering method with delay compensation, and they have relatively slow convergence speed and fluctuate between −0.4° and −0.2° after 175 s. It indicates that it is difficult to accurately estimate the value of *φ_U_* in the process of transfer alignment, so the estimation values of *φ_U_* have some fluctuation ranges, but when using the adaptive H∞ filtering method with delay compensation, the estimation curve of *φ_U_* converges quickly and eventually becomes steady at around −0.025°, so the adaptive H∞ filter with delay compensation has obvious advantage in the estimation of *φ_U_* compared with the other two filtering methods.

**Figure 13:**
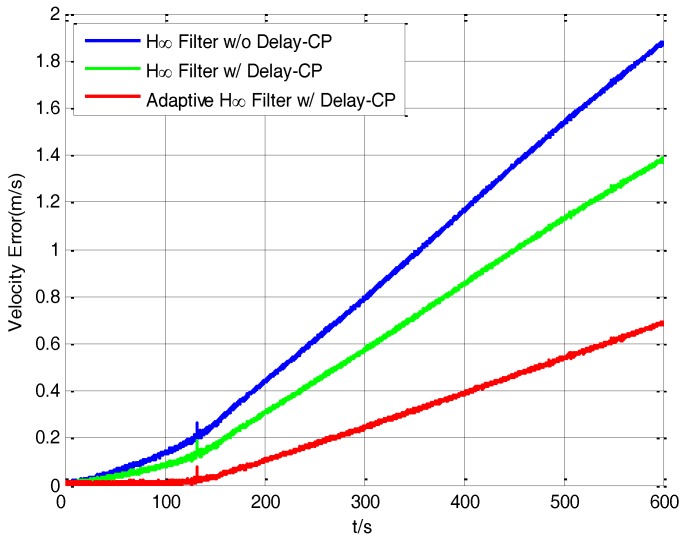
The curves of velocity error in inertial navigation.

In order to verify the transfer alignment accuracy for the three filtering methods, a 10 min’ pure inertial navigation experiment after transfer alignment is conducted. The S-SINS starts pure inertial navigation immediately after 234 s’ transfer alignment, so the navigation accuracy is related to the transfer alignment accuracy. The more accurate the transfer alignment is, the more accurate the pure inertial navigation will be. Figure 13 shows the curves of the velocity error by using the H∞ filtering method without delay compensation, H∞ filtering method with delay compensation, and adaptive H∞ filtering method with delay compensation during the period of S-SINS’s pure inertial navigation.

It can be seen from Figure 13 that the velocity error of the adaptive H∞ filter with delay compensation is the smallest compared with the other two filtering methods. After 10 min’ pure inertial navigation, the velocity errors of the H∞ filter without delay compensation, H∞ filter with delay compensation, and the adaptive H∞ filter with delay compensation are 1.89 m/s, 1.37 m/s and 0.69 m/s respectively.

**Figure 14:**
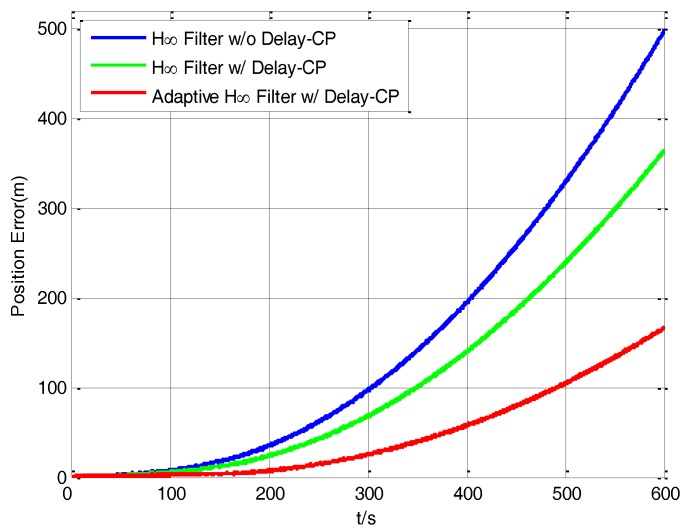
The curves of position error in inertial navigation.

The curves of position errors by using the three filtering methods during the period of S-SINS’s pure inertial navigation are shown in Figure 14. After 10 min’ pure inertial navigation, the position errors of the H∞ filter without delay compensation, H∞ filter with delay compensation and adaptive H∞ filter with delay compensation are 497.93 m, 364.45 m and 166.69 m respectively. The position error of H∞ filter with delay compensation is reduced by 26.82% compared with the position error of H∞ filter without delay compensation. The position error of the adaptive H∞ filter with delay compensation is reduced by 54.23% compared with the position error of the H∞ filter with delay compensation. Totally, the position error of the adaptive H∞ filter with delay compensation is reduced by 66.52% compared with the position error of the H∞ filter without delay compensation, which achieves a good result in the reduction of position error.

**Figure 15:**
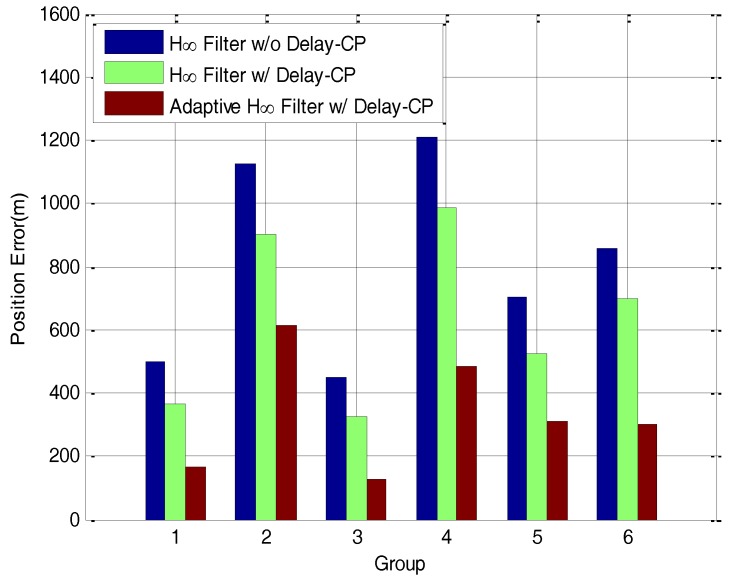
The histograms of position errors in 6 groups of vehicle experiment.

With the purpose of comparing the three mentioned filtering methods in the process of transfer alignment, a total of six groups of vehicle transfer alignment experiments have been conducted. The moving trajectories in the six groups of vehicle experiment are different from each other and the trajectories of the vehicle are random during the transfer alignment experiments. The delay time between M-SINS and S-SINS are different and unknown in all groups of vehicle experiment systems. The changes of vehicle speed and road conditions in the six groups of vehicle experiment are also different from each other. After 6 s’ estimation of time delay and 234 s’ transfer alignment, the vehicle starts 10 min’ pure inertial navigation in all groups of vehicle experiment. The histograms of position errors after 10 min’ pure inertial navigation in 6 groups of vehicle experiment are shown in Figure 15. The position errors in the six groups of vehicle experiments are listed in Table 1.

From Figure 15 and Table 1, the position errors by using the H∞ filter without delay compensation, H∞ filter with delay compensation and adaptive H∞ filter with delay compensation are compared to each other. It can be seen that the position errors by using the H∞ filter without delay compensation are the largest in the six groups of vehicle experiments. All the position errors by using the H∞ filter with delay compensation are smaller than the position errors by using the H∞ filter without delay compensation, but they are larger than the position errors by using the adaptive H∞ filter with delay compensation. In other words, all the position errors by using the adaptive H∞ filter with delay compensation are the smallest in the six groups of vehicle experiments. This indicates that the transfer alignment accuracy by using the adaptive H∞ filter with delay compensation method is higher than the other two methods, which fully demonstrates the advantages of the proposed method.

## 5. Conclusions

In this paper, an adaptive H∞ filtering method with delay compensation for SINS’s transfer alignment is designed, and a transfer alignment model containing a SINS error dynamics model and system measurement model is established. Based on the transfer alignment model, the time delay in the process of transfer alignment is analyzed, and a H∞ filtering method with delay compensation is presented. The H∞ filtering theory with time delay is discussed in detail, and the robust mechanism of H∞ filter is analyzed systematically. In order to improve the performance of H∞ filtering method with delay compensation, an adaptive H∞ filtering method with delay compensation is proposed. In this new method, the robustness factor *ξ* is adjusted adaptively according to the unknown external environment, and the transfer alignment accuracy can be effectively improved.

In order to verify the adaptive H∞ filtering method with delay compensation, a total of six groups of vehicle transfer alignment experiments on moving bases are conducted in real systems. The experimental results show that compared with the H∞ filtering method without delay compensation and H∞ filtering method with delay compensation, the proposed method has the highest transfer alignment accuracy. The research presented in this paper provides a new idea for SINS’s transfer alignment on a moving base.

## Figures and Tables

**Table 1:** The position errors (m) in 6 groups of vehicle experiment.

Method	Group 1	Group 2	Group 3	Group 4	Group 5	Group 6
H∞ Filter w/o Delay-CP	497.93	1126.84	452.31	1210.27	703.42	857.77
H∞ Filter w/Delay-CP	364.45	902.62	323.98	985.92	522.06	696.63
Adaptive H∞ Filter w/Delay-CP	166.69	614.55	128.09	486.46	310.28	302.34
